# Nectar robbing in bellflower (*Sesamum radiatum*) benefited pollinators but unaffected maternal function of plant reproduction

**DOI:** 10.1038/s41598-019-44741-y

**Published:** 2019-06-07

**Authors:** Sangeetha Varma, Palatty Allesh Sinu

**Affiliations:** Present Address: Department of Animal Science, Central University of Kerala, Periya, Kasaragod, 671316, Kerala India

**Keywords:** Behavioural ecology, Behavioural ecology, Biodiversity, Biodiversity

## Abstract

Nectar robbing – foraging nectar illegitimately – has negative, neutral, or positive effects on maternal function of plant reproduction and/or on pollinators. It has been suggested that nectar robbing has a non-negative effect on maternal function of plant reproduction in autogamous and mixed breeding plants; however this hypothesis requires deeper understanding with more studies. We investigated the impact of natural nectar robbing on maternal function of plant reproduction and visitation characteristics of pollinators in *Sesamum radiatum*, an autogamous plant. Pollinators were observed on unrobbed open flowers and robbed open flowers. In robbed flowers, pollinators’ visit type and foraging time were examined. The seed sets of these flower types were examined. *Xylocopa latipes* was both a primary robber and a legitimate pollinator, *X. bryorum* was an exclusive primary robber, and *Megachile disjuncta* was a cosmopolitan pollinator. In robbed flowers, most of the pollinators foraged mostly as secondary nectar robbers. The foraging time shortened considerably when pollinators robbed nectar – a positive effect on pollinators’ foraging efficiency. Robbing did not negatively affect seed set – a neutral effect on the plant’s reproduction. Our study agrees that nectar robbing might have a non-negative effect on reproduction in autogamous and mixed breeding plants.

## Introduction

Plant-pollinator interaction is an example of mutualism. In this interaction, both the plant and the pollinator are benefited from the visits of pollinators on the flowers^1^. However, many plant-pollinator mutualisms are disrupted by cheaters^2^ – visitors that exploit but leave the flowers unrewarded. Nectar robbers are often costly and ubiquitous disruptor of plant-pollinator mutualisms^3,4^. Nectar-robbing is an adaptive trait evolved in some pollinators, which increases their foraging efficiency over legitimate pollinators^4–8^. It has been predicted that facultative exploiters, such as floral nectar foragers, might exploit rather than collaborate with the robbed flowers in order to improve their foraging efficiency^5–7^.

In plant-pollinator-robber interactions, although robbers are always benefited^2^, plants and pollinators may be affected positively or negatively; or remain unaffected^3,8–12^. Like any other species interactions^13^, the net effect of nectar robbing on each partner species and on the plant-pollinator mutualism depends on the context^4^. The breeding mechanism of plants might predict the net effect of nectar robbing on plants’ maternal function of plant reproduction^12,14^.

Nectar robbing in plants can affect male or female reproductive functions^4^. Nectar robbing has a negative effect on female reproductive function in both self-compatible and self-incompatible species which have not developed autogamy as a reproductive strategy^12,14^. Nectar robbing in plants that set seeds through selfing may have a positive or neutral effect on the maternal function of the plant reproduction^8^. Zhang *et al*.^14^ studied the effect of nectar robbing in three sympatric plant species having three different mating strategies (selfing, facultative outcrossing, and obligate outcrossing) and found that the female reproductive function of selfing species was not affected, the facultative outcrossing species was benefited, and the obligate outcrossing species was negatively affected. Similarly, Burkle *et al*.^12^ suggested that nectar robbing has negative effect on female reproductive function of pollen-limited self-incompatible plants. However, testing this hypothesis requires deeper understanding with more case studies.

Nectar robbing may also indirectly affect plant reproduction by affecting the visitation characteristics of pollinators^15,16^. As the nectar-robbed flowers are manipulated, some pollinators may find such flowers less attractive and avoid or reduce their legitimate visitation rate^17–20^. Some pollinators may also reduce the time they spent on the robbed flowers during their legitimate visits^13,21^. Usually, the nectar – a major commodity that attracts legitimate pollinators to the flowers – is manipulated in robbed flowers; and the nectar in robbed flowers may be consumed or evaporated quickly, making the nectar viscous and the flowers less attractive^22^. However, some plants overcome this loss by altering the nectar replenishment pattern in robbed flowers and ensure the visits of legitimate pollinators^23^.

Another consequence of nectar robbing is that some legitimate pollinators bypass the flower opening and access nectar as secondary nectar robbers^4,11,17,24^. The robbed flowers may also open themselves to a new suite of ephemeral visitors if the robbers are not guarding the robbed flowers^24^. Studies also suggest that robbing through the holes made by primary robbers is more economical for the pollinators than using flower opening for nectar efficient nectar foraging^4,23^. True pollinators may improve pollen delivery and cross pollination^25^. However, nectar robbers may not help in plant reproduction directly^26^. Also, since these outcomes are likely to be predicted by the reproductive strategies of plants^12,14^, drawing a general conclusion in this regard needs more consistently harmonious results. Nectar robbing, therefore, has mixed or different outcomes for plants and pollinators.

In the present study, we examined the effect of nectar robbing by two species of carpenter bee – *Xylocopa latipes* and *X. bryorum* – on female reproductive function and pollinators of *Sesamum radiatum* (Pedaliaceae). Unlike many previous studies, the effect of nectar robbing on pollinators in this study was examined on naturally-robbed flowers. Since bees mark the visited flowers, effect of nectar robbing on pollinators’ visits and behaviour can be understood only if the observations are made on naturally-robbed flowers^3^. *S. radiatum* is a wild relative of the oilseed, *S. indicum*, a self-pollinated species. Although selfing has been evolved as a reproductive strategy in most *Sesamum* spp, studies suggest that the bee visits improve pollination^27^. Since the general prediction was that nectar robbing has a non-negative effect on autogamous and facultative-outcrossing species^14^, we hypothesized that nectar robbing in *S. radiatum* may have a neutral effect on maternal function of plant reproduction. We predicted that robbing may a) increase the robbing visits of pollinators in robbed flowers and b) decrease the nectar foraging time of pollinators while robbing. We then measured seed set to assess which direction these predicted changes impacted maternal reproductive function of plant reproduction.

## Methods

### Study site

Fieldwork was conducted in the western valley of the Western Ghats biodiversity hotspot^28^. Sites in Kannur (12°02′8371″N; 75°26′1361″E) and Kasaragod (12°54′8148″N; 75°16′5202″E)) districts of the state of Kerala in the tropical regions of peninsular India were selected. We selected three major locations (Periye, Nileswar, and Pilathara) for the present observational studies in *S. radiatum*. These locations stood at a mean distance of about 11 km apart as the crow files. In each location, we had one to three sites for observations; the sites in a location stood at a mean aerial distance of about 0.7 km. In each site, flowering plants were selected for recording flower visitors and seed set. Detailed methodologies for sampling plants are given below under each objective. The maximum and minimum average temperatures during the study period were 29 °C and 22.82 °C respectively.

### Study species

**Figure 1:**
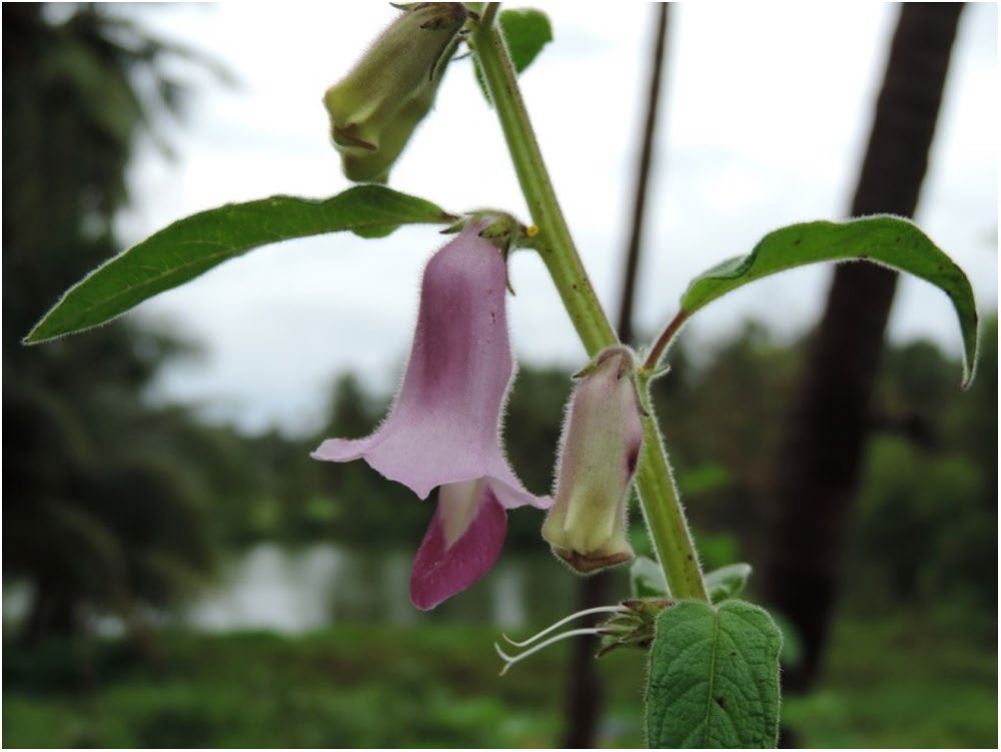
A flower of *Sesamum radiatum*.

*Sesamum radiatum* is an annual herb that attains a maximum height of about 1.5 metres on both sandy and laterite rocky soils. It produces deep pink bell-shaped tubular hermaphroditic flowers on leaf axils (Fig. 1), which is open only for a day. The corolla tube is made up of five fused petals; one petal has an extended lip, which is used by the legitimate insect pollinators to land and enter the flower from front. The androecium has four didynamous anther filaments (two 1.7-cm long and two 1.1-cm long), which are attached on the inner wall of the corolla tube opposite to the extended lip. The anthers dehisce longitudinally. The gynoecium with a style of length 1.7 cm and two-lobed stigma passes in between the anther filaments and opens at the level of longest anther filaments. This arrangement of sexual parts may facilitate autogamy (Varma and Sinu unpubl.).

Although *Sesamum* spp including the domesticated *S. indicum* are self-pollinated^27^, the reproductive strategy of *S. radiatum* was unknown before our study. We observed carpenter bees, honey bees, solitary bees, wasps, and butterflies foraging both the nectar and pollen from *S. radiatum* flowers (Table 1). *X. latipes* and *X. bryorum* were the dominant primary robbing visitors of *S. radiatum*. *Megachile disjuncta* was the cosmopolitan pollinator of the flowers.

**Table 1:** Flower visitors and their functions in robbed and unrobbed flowers of *Sesamum radiatum*.

Visitor species	Robbed flower			Unrobbed flower
	Family	Legitimate	Secondary Robber	Legitimate
*Amegilla singulata*	Apidae	√	√	—
*Amegilla zonata*	Apidae	√	√	—
*Apis dorsata*	Apidae	—	—	√
*Ceratina hieroglyhica*	Apidae	—	√	√
*Ceratina smaragdula*	Apidae	—	√	√
*Xylocopa acutipennis*	Apidae	—	√	—
Xylocopa bryorum*	Apidae	—	√	—
Xylocopa latipes*	Apidae	√	√	√
*Nomia curvipes*	Halictidae	—	—	√
*Nomia iridescens*	Halictidae	—	√	√
*Lasioglossum sp*	Halictidae	—	√	√
*Megachile disjuncta*	Megachilidae	√	√	√
*Megachile fulvovestita*	Megachilidae	—	—	√
*Megachile sp*	Megachilidae	√	√	√
*Chelostoma sp*	Megachilidae	—	√	√
*Campsomeriella collaris*	Scolidae	—	—	√
*Anterhynchium abdominale*	Vespidae	—	—	√
*Hesperidae sp1*	Hesperidae	—	—	√
*Catopsilia pomona*	Pieridae	—	—	√
*Delias eucharis*	Pieridae	√	√	—
*Graphium agamemnon*	Papilionidae	—	—	√
Hawkmoth	Sphingidae	—	—	√

*Primary nectar robber.

### Effect of nectar robbing on pollinators

Surveys of nectar robbing in ten sites (151.6 ± 27.98 flowers/site) revealed a mean robbing rate of 61.91 ± 13.11% (±SE; range = 0–97.87%; median = 82.74%) of flowers per site. Nectar robbing rate in seven sites was consistently higher (85.71 ± 5.09%) than the other three sites (4.4 ± 3.86%; F_1,481_ = 1476, p < 0.0005). The heavily robbed sites (N = 7) were also different on the proportion of robbed flowers per plant (F_6,239_ = 7.38, p < 0.0005).

In each site we watched flowers soon after anthesis (approx. 0800 h) on clear sunny days for one hour (normally between 0800 h–0900 h) during the peak flowering period. The observer stood at a convenient spot that allowed the observer to record the visits on a convenient number of flowers. The numbers of observed plants and flowers varied between three and twenty-six and 20 and 169, respectively. We recorded visitors to all flowers of the selected plants. We recorded visitor species, number of visits, and mode of visit (robbing or legitimate) for every visits. Because we used different numbers of flowers across sites for the observation, we used the number of visits and the number of flowers to calculate visitation rate. Visitation rate of a species in a given site, expressed in terms of visits/flower^−h^, is therefore calculated by dividing the total number of visits made by that species on the focal flowers of that site by the number of focal flowers watched in that site. We used the same method to find out legitimate and nectar robbing visitation rates. For some visits of pollinators and robbers, we also recorded the time spent/visit.

Although the frequency of robbed flowers in three sites was very low, the effects of nectar robbing on pollinators and plant reproduction were studied in robbed and unrobbed flowers of all the ten sites. Like other visitors, the primary nectar robbers also made their robbing visits soon after anthesis. Therefore, we caged flower buds the previous evening using paper bags to limit the visitors in flowers before our arrival. At around 0800 h, we opened the bags and allowed visitors. At the beginning of our observations all flowers were unrobbed. As soon as we saw a robbing visit in a flower, we labelled that flower as primary nectar robbed flower and observed for one hour from then. The flowers that had no robbing visits for one hour of observation were considered as unrobbed open pollinated flowers. These flowers were revisited before senescing to ensure that they were not robbed after our observation hours. All the observations on robbed and unrobbed flowers were completed within two hours from anthesis. We collected voucher specimens of all flower visitor species, identified the genus and then to species or morphospecies, and deposited in the Entomology collection of Insect museum of Central University of Kerala.

For the present study, visitors that visited flowers from the front are considered as pollinators and those that used nectar hole created by primary nectar robber as robbers. In robbed flowers the visits from the front of the flower are considered as legitimate visits and the visits through nectar hole are considered as robbing visits. Therefore, when a primary robber accessed a flower from front, the visit was considered as a legitimate visit. Simultaneously, when a pollinator accessed a flower from calyx side, it was considered as a robbing visit. First, we compared the visitation rates of overall visitors in robbed and unrobbed flowers across sites to understand whether the robbing had any effect on overall flower visitors. We analyzed the visitation data using linear mixed effect model (function lmer in package lme4^29^ in R 3.2.3). We considered visitation rate as the response variable and flower type as fixed effect and sites nested within location as random variable. Then we restricted our observations on robbed flowers and studied the visit type (legitimate visit and robbing visit) of pollinators. We analyzed the visitation rate data of the two visit types using linear mixed effect model (function lmer in package lme4). We considered the visitation rate as the response variable, visit type as fixed effect, and sites nested in location as the random effect. We recorded the flower handling time of visitors during their robbing and legitimate visits and analyzed the data using linear mixed effect model (function lmer in package lme4). We considered flower handling time as the response variable, visit type as fixed effect, and flower ID nested within plant ID within location as the random effect.

### Effect of nectar robbing on maternal function of plant reproduction

We had two types of flowers for studying the impact of nectar robbing on seed set: robbed open flowers and unrobbed open flowers; the robbed open flowers had robbing visit first and subsequent legitimate and secondary robbing visits and the unrobbed open flowers had only legitimate visits. We studied a third category of flower – unrobbed caged flowers – which shuts off for all visitors; this is used to investigate the impact of caging on seed set in *S. radiatum*. We harvested fruits on the 10^th^ day of tagging flowers, opened the fruit capsules, and counted the number of seeds. To assess the impact of nectar robbing on maternal function of plant reproduction, we used seed set per capsule as the response variable. Although we collected data on fruitset, we did not use it as many fruits despite had developed normal capsules had no seeds in them. However, there were no signs of seed predation on fruit capsules. Assuming that the number of available fruit capsules on plants may have a negative feedback on subsequent fruit setting^30^, we recorded the number of fruit capsules present on plants on the day of tagging flowers and used it as a random factor in the model. We used linear mixed effect model (function lmer, package lme4) to study the effects of nectar robbing on the mean seed set per plant with number of available fruit capsules nested within plants within sites within location.

## Results

### Effect of nectar robbing on pollinators

The flowers of S. *radiatum* attracted 22 visitors (fifteen bees including the two primary robber species, five Lepidoptera including one hawk moth and four butterfly species, and two wasp species). Among the two species of primary nectar robbers – *Xylocopa latipes* and *X. bryorum* – the former was both a pollinator and a nectar robber, but the latter was an exclusive nectar robber. Both the species made only one hole at the nectary/ovary level of the corolla tube of pendent *S. radiatum* flowers. Among the remaining twenty floral visitors, *Megachile disjuncta* was a cosmopolitan dominant pollinator of *S. radiatum*.

**Figure 2:**
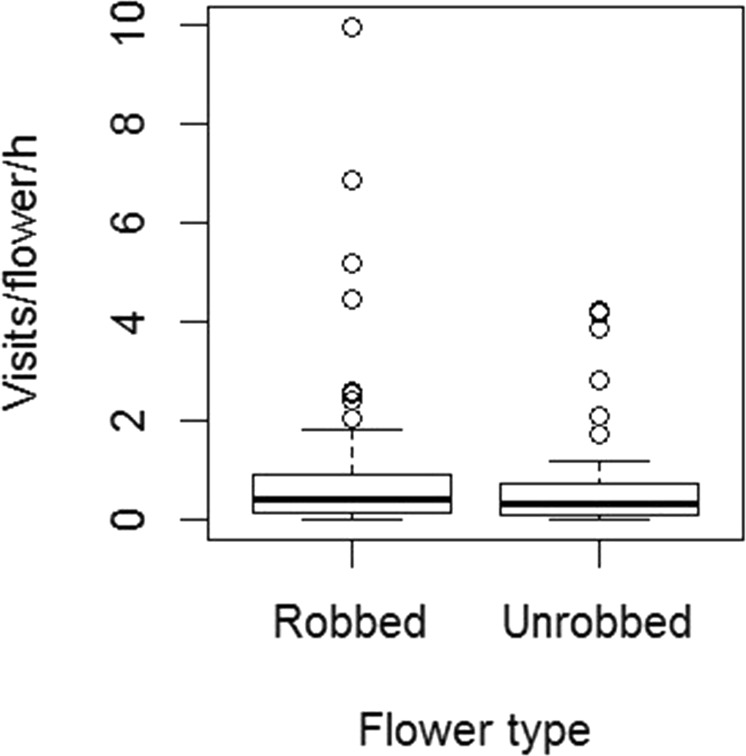
Nectar robbing had no effect on visitation rate of overall flower visitors of *Sesamum radiatum* (N_obs_ = 199).

**Figure 3:**
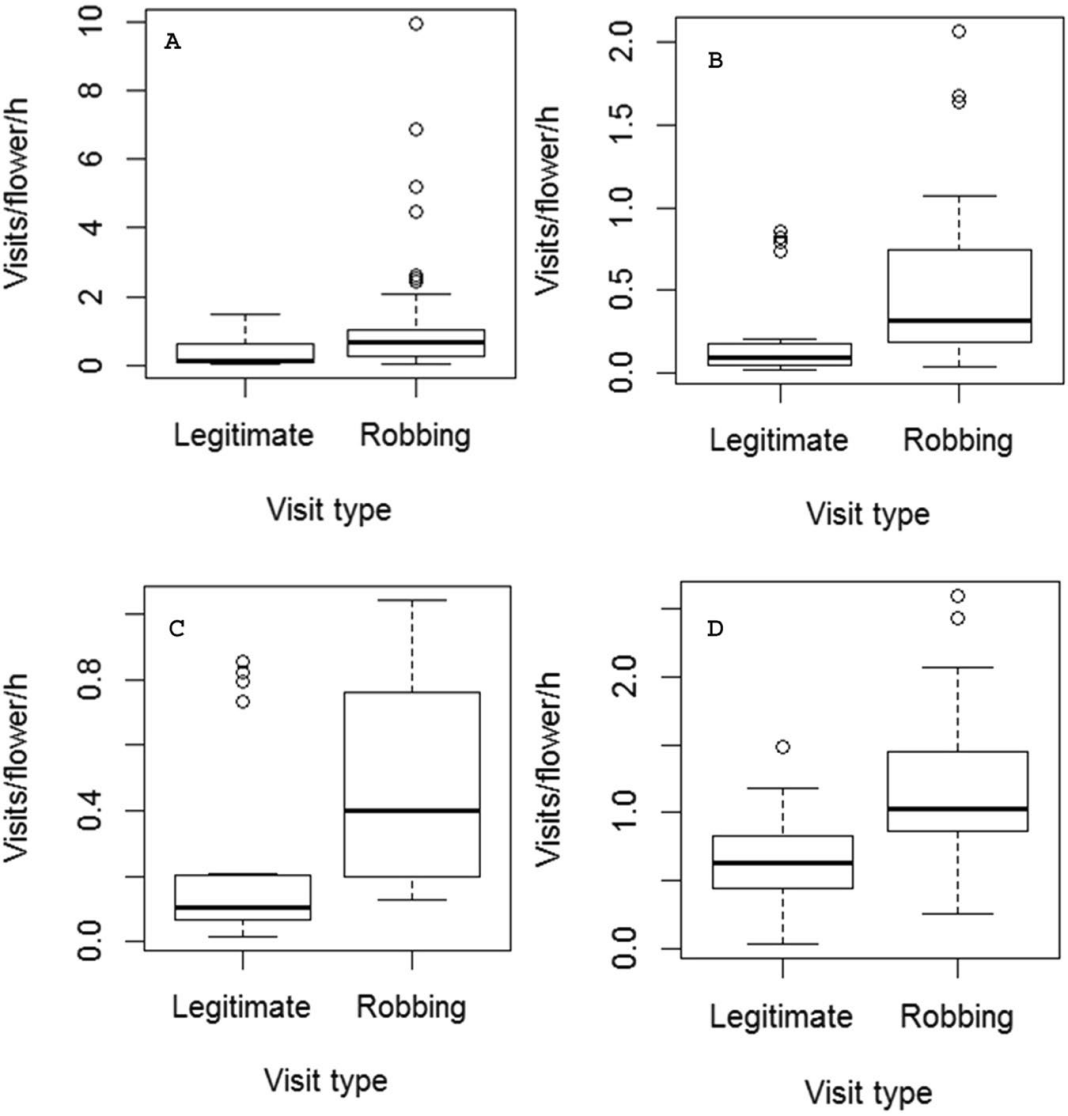
Nectar robbing had a negative effect on legitimate visits of (**A**) overall visitors (N_obs_ = 144), (**B**) pollinators (N_obs_ = 97), (**C**) *Megachile disjuncta* – a cosmopolitan pollinator of *Sesamum radiatum* (N_obs_ = 59), and (**D**) *Xylocopa latipes* – a primary nectar robber cum pollinator of *Sesamum radiatum* (N_obs_ = 39).

Although the proportion of robbed flowers of seven heavily robbed sites was different, the visitation rate of pollinators on robbed and unrobbed flowers of those sites was not different (F_6,17_ = 0.385, p = 0.87); but, that on robbed flowers of those seven sites was different (F_5,8_ = 4.37, p = 0.03). Robbing had no effect on richness (robbed flowers = 13; unrobbed flower = 16; Chi-square = 0.03, d.f. = 1, p = 0.8) and visitation rate (slope ± SE = −0.20 ± 0.19; t = −1.04, df = 179, p = 0.29) of overall floral visitors in robbed flowers (Fig. 2). The species richness of legitimate visitors of robbed and unrobbed flowers were not different (robbed flowers = 10; unrobbed flower = 15; Chi-square = 1.00, d.f. = 1, p = 0.31). The richness of legitimate visitors and secondary nectar robbers of robbed flowers were also not different (legitimate visitors = 10; secondary nectar robbers = 13; Chi-square = 0.39, d.f. = 1, p = 0.53). However, when we examined the robbing and legitimate visitation rates on robbed flowers, we found that the robbing visits of overall visitors (slope ± SE = 0.53 ± 0.19; t = 2.73, df = 143, p = 0.007), pollinators (slope ± SE = 0.31 ± 0.07; t = 4.00, df = 95, p = 0.0001), and *M. disjuncta* (slope ± SE = 0.24 ± 0.08; t = 3.05, df = 56, p = 0.003) had increased over their legitimate visits. *X. latipes* – the primary robber cum pollinator – also had made more number of robbing visits than the legitimate visits (slope ± SE = 0.61 ± 0.19; t = 3.29, df = 15, p = 0.004) (Fig. 3).

**Figure 4:**
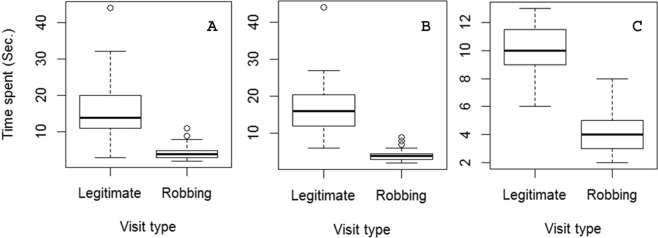
Nectar foraging time of (**A**) overall pollinators (N_obs_ = 140), (**B**) *Megachile disjuncta* (N_obs_ = 83), and (**C**) *Xylocopa latipes* –the robber cum pollinator (N_obs_ = 27) – decreased drastically when they robbed nectar.

The foraging time of overall pollinators (slope ± SE = −12.61 ± 1.00, t = −12.55, df = 127, p < 0.00005), *M. disjuncta* (slope ± SE = −13.8 ± 1.32, t = −10.4, df = 81, p < 0.00005), and *X. latipes* (slope ± SE = −6.05 ± 0.55, df = 23, t = −11.03, p < 0.00005) decreased drastically when they robbed nectar as secondary nectar robbers (Fig. 4).

### Effect of nectar-robbing on maternal function of plant reproduction

**Figure 5:**
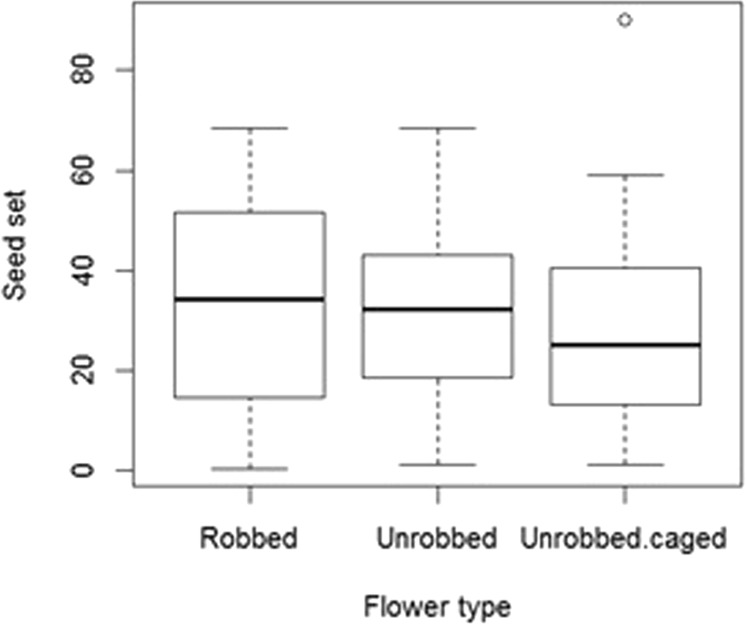
Nectar robbing had no significant effect on seed set, but caging had a negative effect on seed set (N_obs_ = 165).

Like the proportion of robbed flowers differed significantly between the seven heavily-robbed sites, the seed set of those sites also differed significantly (F_6,51_ = 4.98, p = 0.0004). The robbing had no significant effect on seed set (slope ± SE = 8.27 ± 4.74; t = 1.74, df = 27, p = 0.09). The caging of flowers had a significant negative effect on seed set (slope ± SE = −7.51 ± 2.71; t = −2.77, df = 157, p = 0.006). The results suggest that pollinator visits improve seed set in *S. radiatum*, but robbing does not affect seed set (Fig. 5).

## Discussion

The present study on naturally-occurring plant-robber interaction in *Sesamum radiatum* – the wild relative of oilseed, *Sesamum indicum* – in tropical India suggests that robbing has no significant effect on maternal function of plant reproduction despite having some impact on the visitation characteristics of pollinators. Studies have reported that nectar robbing has positive, negative, and neutral effects on the maternal function of plant reproduction (see a review^4^ and references therein). Our results also agree with the previous studies that show nectar robbing has mostly either neutral or positive effect on maternal function of plant reproduction in autogamous and facultative outcrossing species^12,14,24^.

Identified mechanisms of nectar robbing which have negative effects on maternal function of plant reproduction include damaging ovary and other reproductive structures of flowers while probing for flowers at nectary level^31^, aggressively interacting with the pollinators^32^, and/or making the robbed flowers unattractive to pollinators^17,24^. The robbed flowers will be unattractive to some pollinators when the nectar profile of flowers is affected^17,24^ or when the flower morphology is considerably mutilated for a pollinator to visit (Varma *et al*. (under review)). In our study the robber seemed not to have an aggressive interaction with the pollinators. The pollinators maintained both the legitimate and robbing visits on robbed flowers. The flower was also not mutilated badly; therefore the legitimate visits were also maintained on robbed flowers.

There are multiple mechanisms that suggest that nectar robbing has non-negative effects on the maternal function of plant reproduction in autogamous and facultative outcrossing plants. One hypothesis suggests that the pollinators do not distinguish between robbed and unrobbed flowers, and hence maintain their legitimate visits on robbed flowers and pollinate the flowers^3,33^. The second hypothesis is that the pollinators do distinguish robbed flowers from unrobbed flowers, and that they visit robbed flowers as secondary nectar robbers^14,24,34,35^; however, during their robbing visits they load the stigma with pollen. Our result that nectar robbing has no negative effect on plant reproduction in *S. radiatum* might have been partly due to the second mechanism explained here.

Mating system in plants also predicts the direction of the effect of nectar robbing on plants’ maternal reproductive function^12,14,24^. It is predicted that self-incompatible plants experience negatively in terms of fruit set or seed set and self-compatible plants have a non-negative effect on maternal function of plant reproduction. In an Andean self-compatible tree, *Oreocallis grandiflora*, nectar robbing, despite caused a drop in the frequency of pollinators on robbed flowers, had no effect on seed set or seed mass^24^. In an alpine self-compatible plant, *Salvia przewalskii*, because the nectar resecretion allowed legitimate visits of pollinating bumblebee on robbed flowers, the fruit set and seed set of robbed flowers were not affected^23^. In congruence with these studies^23,24^, we also found that nectar robbing had not significantly affected seed set. The stigma of *S. radiatum* flower opens at the level of two longest anther filaments. Therefore subtle vibrations on flowers can transfer pollen grains to stigma. Since *S. radiatum* is benefited from autogamous pollination, robbing visits might have allowed the anthers to liberate pollen grains and facilitate self-pollination in robbed flowers. The seed set data of caged flowers also suggests that the plant is benefited from the visits of legitimate visitors. The seed set of robbed open flowers was not different from that of unrobbed open flowers. The robbing had no effect on the visitation rate of overall pollinators on robbed flowers. Both the pollinators and the primary robber – *X. latipes* – maintained some legitimate visits in robbed flowers. Additionally, the species richness of legitimate visitors of robbed flowers was not considerably affected by nectar robbing. These visitors might have facilitated both the self and cross pollination in the robbed flowers.

Nectar robbing in *S. radiatum* had a positive effect on pollinators’ foraging efficiency. The pollinator richness did not differ significantly between robbed and unrobbed flowers; however, the foraging behaviour of pollinators is changed by nectar robbing. It seems nectar robbing had improved the nectar foraging efficiency of pollinators, holding the predictions of foraging theory^5^. It has been predicted that facultative exploiters, which often lack traits required for primary robbing, may choose secondary robbing strategy if not controlled, because this can improve their nectar foraging efficiency^5–7^. Lichtenberg *et al*.^7^ found that *Bombus mixtus* visiting *Corydalis caseana* more frequently as a robber than a legitimate pollinator. Ye *et al*.^23^ found that the robbing visits of *Bombus friseanus* were considerably shorter than its legitimate visits in an Alpine plant, *Salvia przewalskii*. Both the studies suggest that exploitation yields these bees higher net benefits than collaborating. In our study, pollinators in general and *M. disjuncta* – a cosmopolitan pollinator of *S. radiatum* – in particular became exploiters than collaborators in robbed flowers. The nectar foraging time was considerably low when pollinators robbed nectar as secondary nectar robbers. In addition to this, at least six species of bees (*Ceratina hieroglyphica, C. smaragdula, Chelostoma sp, Nomia iridescens*, *Xylocopa acutipennis*, and *Lasioglossum* sp) foraged robbed flowers exclusively as secondary nectar robber, holding the predictions of mutualism and foraging theories^5^. As juxtaposed with the previous results, *Apis dorsata* selectively visited only unrobbed flowers of *S. radiatum*; it made 2.82 (±0.16) visits/flower^−h^. *A. dorsata* therefore might have distinguished unrobbed flowers from the robbed flowers; however, no visits on robbed flowers prevented us statistical testing separately.

Cases where nectar robbing had no net negative effect on visitation rate of pollinators have a sustained nectar replenishment pattern in robbed flowers^23,36^. Although we did not examine the nectar dynamics of robbed and unrobed flowers in the present study, the sustained visits of pollinators, despite as robbers, on robbed flowers suggest that nectar robbing in *S. radiatum* is unlikely to have a negative effect on nectar replenishment pattern or on quality of nectar in robbed flowers. However, future studies may shed some light on this. We observed flowers for one continuous hour from the time of anthesis to record the visits of pollinators and robbers in unrobbed flowers. Although we started our observation from 0800 h, it continued till noon as the one hour observation on robbed flowers had started from the time when they were robbed. Therefore, it is likely that we recorded most of the flower visitors and captured their temporal foraging behaviour in the flowers of *S. radiatum* in this study.

Studies examining the effect of nectar robbing on pollinator behaviour in naturally-robbed flowers are rare^3^. Artificially robbed flowers – creating a hole and removing nectar manually using a syringe – although useful for studying the effect of nectar robbing on various aspects of pollinators^17,21,23,24^, has limitations as well^3^. Bees may scent-mark the visited flowers which can cause changes in the behaviour of conspecific and heterospecific pollinators^37,38^. It is also applicable in robber-pollinator interactions^3^. The studies that make observations on naturally-robbed flowers can only allow for the likely role of scent marking of robbers and discern the effect of nectar robbing on pollinators’ foraging behaviour^3^. All robbed flowers that we monitored in the present study were done by carpenter bees.

In brief, nectar robbing in *S. radiatum* had a positive effect on pollinators’ foraging efficiency. However, nectar robbing had not affected seed set in the plant. This might be due to the fact that the sustained legitimate visits and the secondary nectar robbing visits of both the pollinators and *X. latipes* – the robber species – in robbed flowers might have facilitated both the self- and cross-pollination. *Sesamum* spp despite are autogamous, cross-pollination contributes to a small proportion of overall pollination^27^.

Studies examining the effect of nectar robbing on plants in tropical environments are relatively very rare (but see, González-Gómez and Valdivia^39^). It is not clear whether the natural plant-robber interaction is rare in tropics or less studied. However, plant-pollinator network is complex in tropics, where robbers can destabilise the pollination system of a species or a community^7,40,41^. Our study might give an insightful understanding of nectar robbing in tropical plants and may prompt researchers to find more such interesting cases in old-world tropics.
